# Supplementary material for: Double k-Grid Method for Solving the Bethe-Salpeter Equation via Lanczos Approaches
Source: Front Chem. 2022 Jan 20;9:763946. doi: 10.3389/fchem.2021.763946 (PMC8811451; doi:10.3389/fchem.2021.763946)
Supplement: Supplementary file 1 [file DataSheet1.PDF]

Supporting Information

Double k-Grid Method for Solving the Bethe-Salpeter Equation via Lanczos Approaches

Ignacio M. Alliati,[1] Davide Sangalli,[2,*] and Myrta Grüning[1,*]

[1]*School of Mathematics and Physics, Queen's University Belfast, Belfast BT7 1NN, Northern Ireland, United Kingdom*

[2]*Istituto di Struttura della Materia—Consiglio Nazionale delle Ricerche (CNR-ISM), Division of Ultrafast Processes in Materials (FLASHit), Via Salaria Km 29.5, CP 10, I-00016 Monterotondo Stazione, Italy*

I. INTERMEDIATE DOUBLE GRIDS

A. Si bulk SI

See Figs. S1 and S2.

B. GaAs bulk SI

See Figs. S3 and S4.

C. MoS_2 monolayer SI

See Figs. S5 and S6.

*Also at European Theoretical Spectroscopy Facility (ETSF)

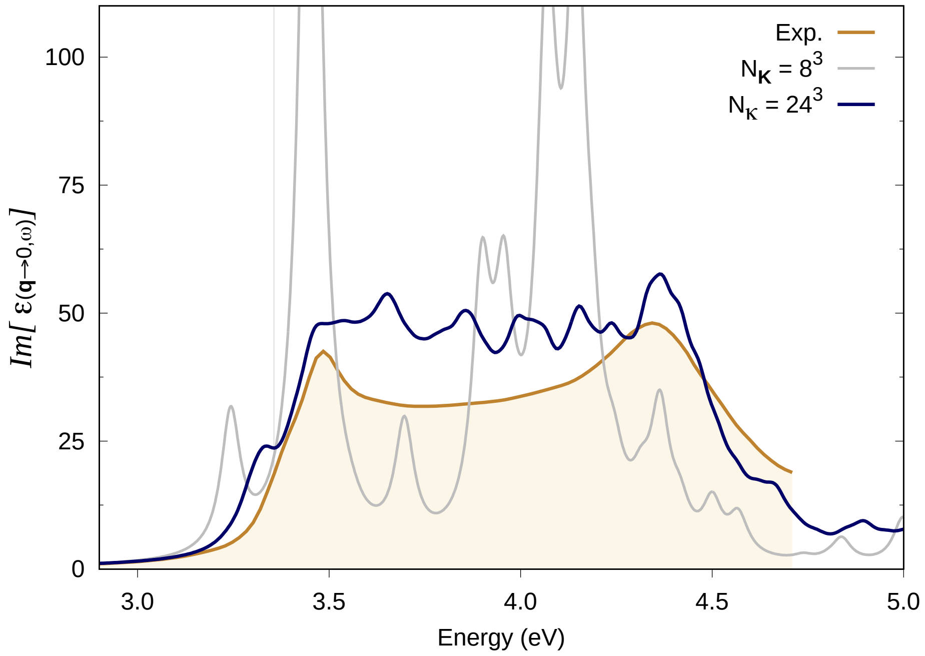

FIG. S1: Optical spectra of bulk Si as in Fig. 1 with a $24 \times 24 \times 24$ double grid

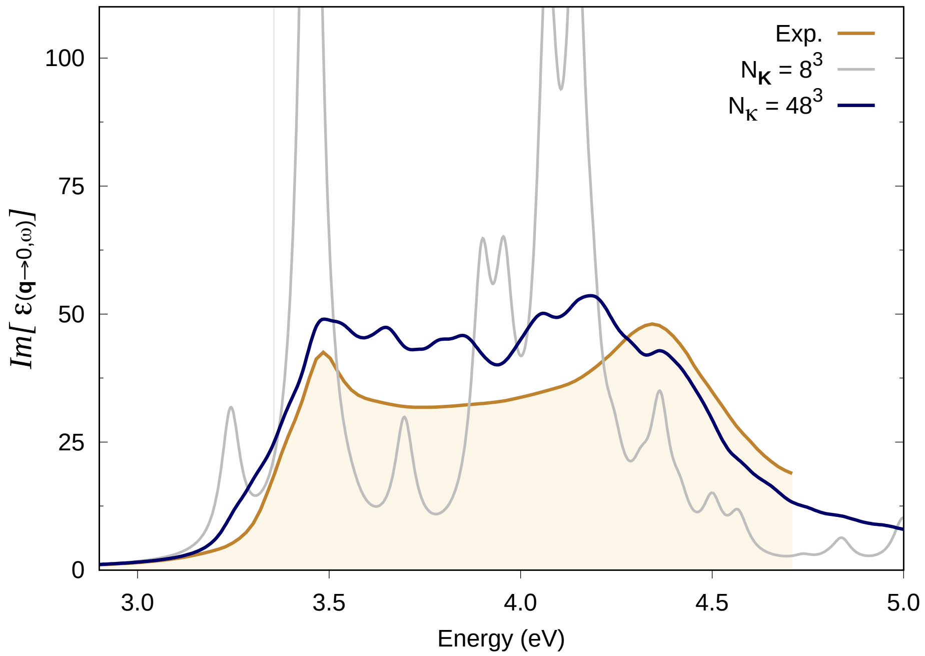

FIG. S2: Optical spectra of bulk Si as in Fig. 1 with a $48 \times 48 \times 48$ double grid

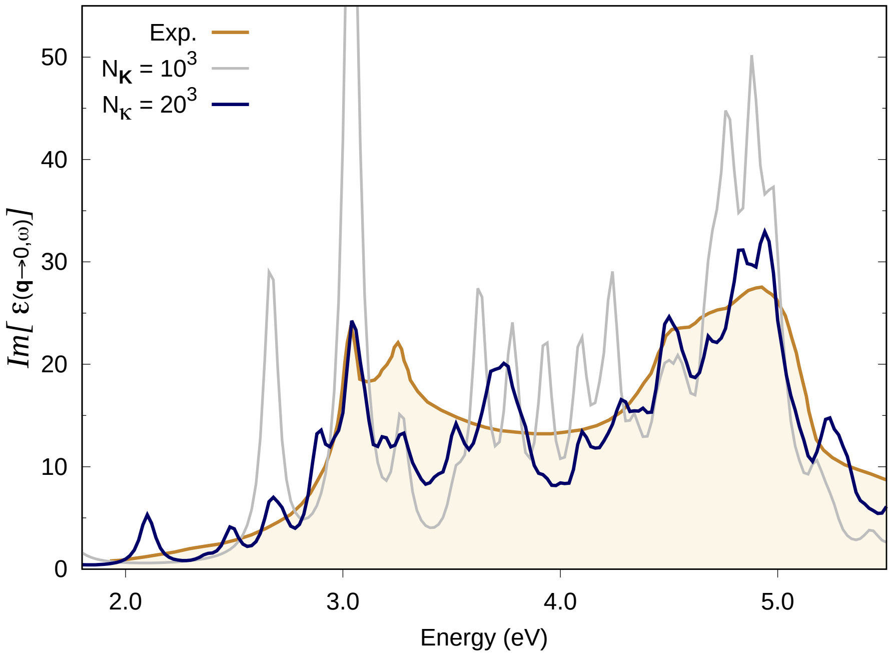

FIG. S3: Optical spectra of bulk GaAs as in Fig. 2 with a $20 \times 20 \times 20$ double grid

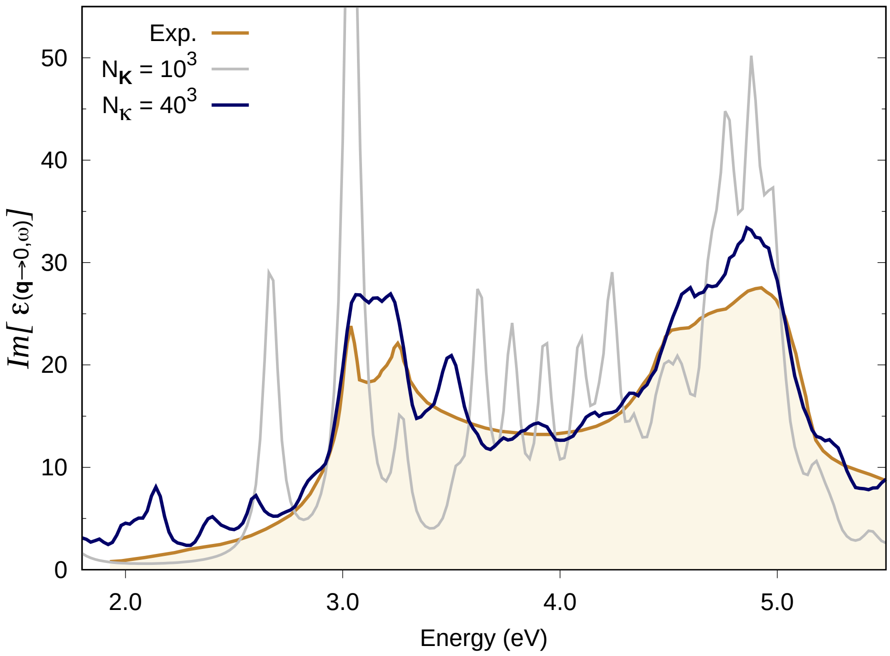

FIG. S4: Optical spectra of bulk GaAs as in Fig. 2 with a $40 \times 40 \times 40$ double grid

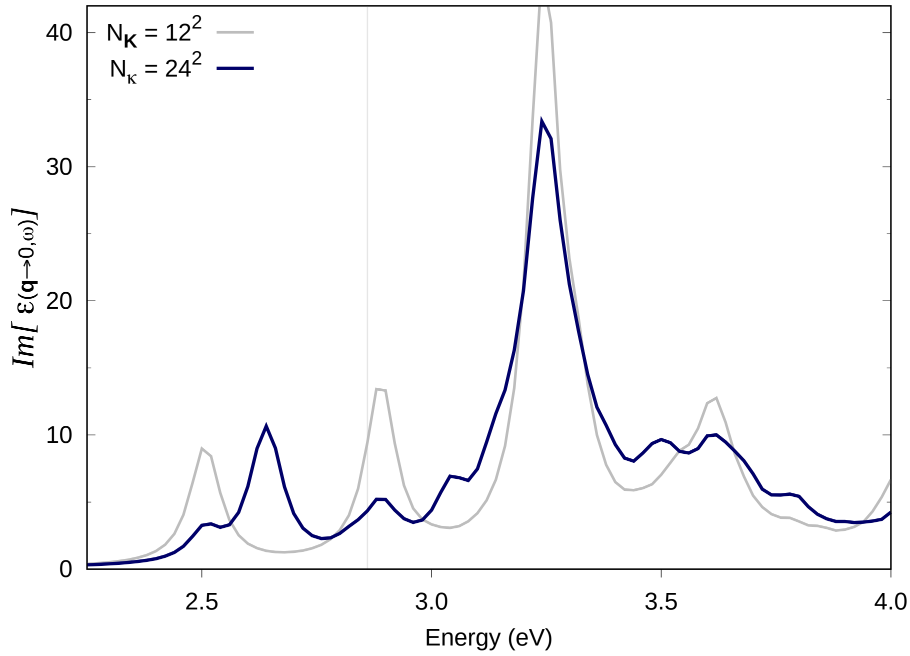

FIG. S5: Optical spectra of monolayer MoS_2 as in Fig. 3 with a $24 \times 24 \times 1$ double grid

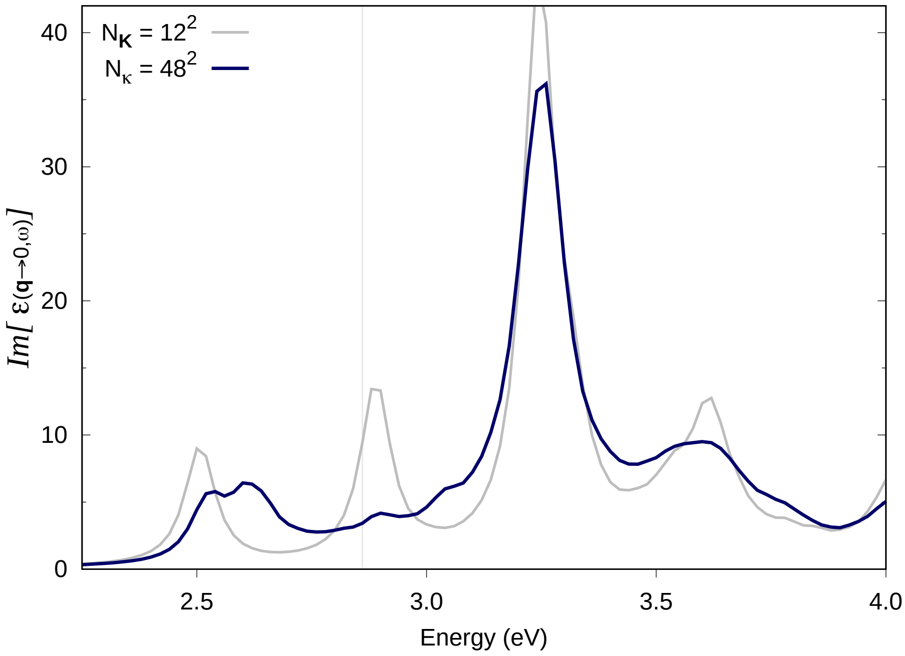

FIG. S6: Optical spectra of monolayer MoS_2 as in Fig. 3 with a $48 \times 48 \times 1$ double grid

II. SHIFTED GRIDS

While Gamma-centred grids have been used throughout the study, our method can also be used with shifted grids, both with regular and random shifts. Taking the case of GaAs as an example, we present the results obtained with regular shifted grids in Fig. S7. The $20 \times 20 \times 20$ shifted fine grid entails 36000 **k**-points, while the $10 \times 10 \times 10$ shifted grid, only 4000. Despite this difference, the two calculations in Fig. S7 took essentially the same time (relative difference less than 1%). This proves that the numerical advantages of the double-grid method are also present in the case of shifted grids.

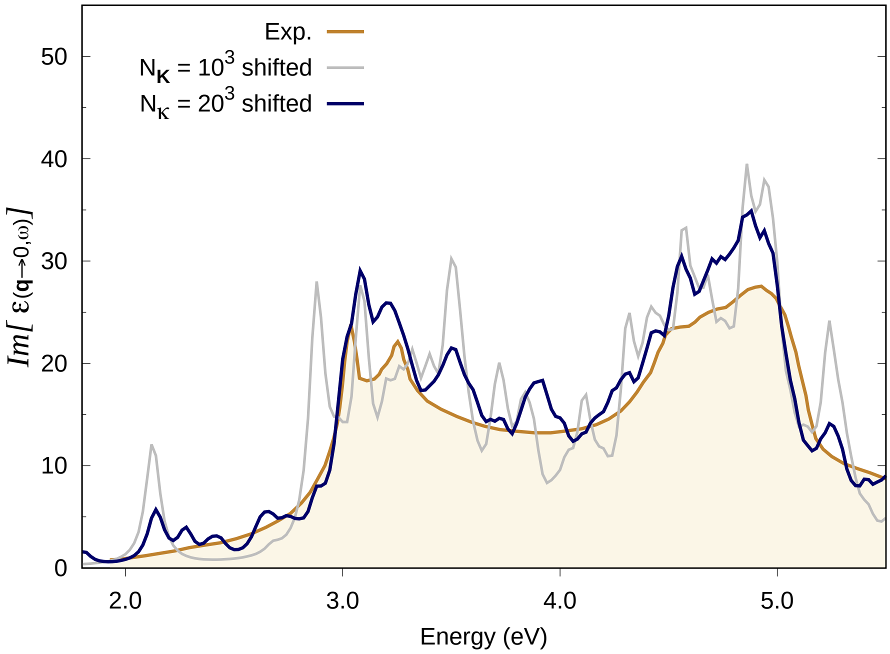

FIG. S7: Optical spectra of bulk GaAs as in Fig. S3 with shifted grids

Regarding the result itself, Fig. S7 shows that shifted grids converge faster, as reported elsewhere in the literature (see for example [1, 2]). In particular, the $10 \times 10 \times 10$ shifted grid result in Fig. S7 looks better converged than the Gamma-centred one in Fig. S2. The same applies to the $20 \times 20 \times 20$ results. However, this is partly due to the fact that shifted grids represent many more **k**-points than Gamma-

centred ones. For instance, Gamma-centred grids of the same dimensions contain 1000 and 4000 **k**-points, respectively. Moreover, there is a subtlety about what high symmetry **k**-points are included when using Gamma-centred or shifted grids, which has implications as to how well a a given grid represents the physics of the material at hand. For example, the direct gap in GaAs (and Si) occurs at the Gamma point, which makes Gamma-centred grids a wise choice in this case.

Finally, the use of random shifted grids on their own right (rather than via a double grid approach) is a well-known method of improving **k**-points convergence. This usually offers faster convergence than an equally dense Gamma-centred grid, while keeping the size of the BSE kernel unchanged. Despite this advantage, the number of **k**-points in the Irreducible Brillouin Zone increases, and so does the memory required to load the corresponding KS orbitals. These increased memory and disk storage requirements could become prohibitively large in materials for which very dense **k**-grids are needed. The random shift method does not address these problems, which are precisely what we intend to target with our method (as the fine-grid KS orbitals are not needed). Furthermore, **k**-grids with random shifts usually do not have the symmetries of the system, with the potential for breaking degeneracies and turning dark excitons into bright ones.

III. CODE AND DATA AVAILABILITY

A. Code availability

The double-grid method developed in this work for calculating optical absorption spectra via the Haydock solution scheme of the BSE will be available in the next release of the Yambo code (Yambo 5.1). A tutorial will be made available in due course through the tutorials section (`http://www.yambo-code.org/wiki/index.php?title=Tutorials`) of the official Yambo website (`http://www.yambo-code.org/`).

B. Data availability

Input and output files of the calculations presented in this study can be found in a GitHub repository (`https://github.com/aim137/double_grid_data_repository.git`)

IV. KERNEL MATRIX IN THE DOUBLE GRID METHOD

As discussed in Section IV B of the manuscript, we explored two possible approaches for the extension of the BSE kernel from the coarse grid to the fine grid, namely, DKE and FKE. The DKE approach was chosen for the double grid Haydock implementation in the Yambo code as it has shown to consistently produce better results than the FKE method. In this Section, we will give a visual representation of the equations defining both approaches. Further arguments in favour of DKE are also presented here, apart from the **q**-dependence of the kernel matrix elements (already explored in Section IV B).

A. Coarse grid

For the sake of argument, let us assume we have a system with two bands (c and v) and two **k**-points ($\mathbf{k_1}$ and $\mathbf{k_2}$). This system would then have two transitions (T_1 and T_2). In this example, the e-h basis would have two components and the BSE kernel would be a 2-by-2 matrix,

$$\begin{bmatrix} \mathbf{\Xi_{11}} & \mathbf{\Xi_{12}} \\ \mathbf{\Xi_{21}} & \mathbf{\Xi_{22}} \end{bmatrix} . \tag{S1}$$

We will also assume that these transitions do not belong in the same *group*, i.e., we define two groups of transitions ($BS\_nT\_grps = 2$, namely T_gr1 and T_gr2). It is according to these groups that the Haydock vector is then divided into fragments. In a realistic case, the number of groups will be given by the number of **K**-points in

the IBZ times the number of cores for the e-h space. In order to work with the BSE matrix, we break it down in *blocks* according to the number of transition groups. In our case, $BS\_nT\_grps = 2$ so there would be 4 blocks, i.e., $(BS\_nT\_grps)^2$ given that each block is defined by two transition groups — In reality, only 3 distinct *blocks* are defined, however that is not relevant to this discussion.

The readily implemented Haydock algorithm requires matrix vector multiplications of the kind

$$\begin{bmatrix} \mathbf{\Xi_{11}} & \mathbf{\Xi_{12}} \\ \mathbf{\Xi_{21}} & \mathbf{\Xi_{22}} \end{bmatrix} \cdot \begin{bmatrix} \mathbf{Vi_1} \\ \mathbf{Vi_2} \end{bmatrix} = \begin{bmatrix} \mathbf{Vo_1} \\ \mathbf{Vo_2} \end{bmatrix}. \tag{S2}$$

B. Double grid

We now add a fine $\mathbf{K}$-grid to our calculation. For the sake of argument, let us say that $\mathbf{K_1}$ has one fine grid $\mathbf{k}$-point associated to it (κ_{1a}), while $\mathbf{K_2}$ has two (κ_{2a} and κ_{2b}) — we use a and b for the numbers associated with the indices $i = 2$ and $i = 3$ in Eq. 12 of the manuscript to help the visual impact of this section. In this case, our e-h basis will grow from having 2 transitions, to 5 in total. The number of transition groups will stay the same, i.e., $BS\_nT\_grps = 2$. The transitions at $\mathbf{K_1}$ and κ_{1a} will still be in the first group, while the transitions at $\mathbf{K_2}$, κ_{2a} and κ_{2b} will form the second group. This will lead to the BSE matrix

$$\begin{bmatrix} \mathbf{\Xi_{11}} & \Xi_{11a} & \mathbf{\Xi_{12}} & \Xi_{12a} & \Xi_{12b} \\ \Xi_{1a1} & \Xi_{1a1a} & \Xi_{1a2} & \Xi_{1a2a} & \Xi_{1a2b} \\ \mathbf{\Xi_{21}} & \Xi_{21a} & \mathbf{\Xi_{22}} & \Xi_{22a} & \Xi_{22b} \\ \Xi_{2a1} & \Xi_{2a1a} & \Xi_{2a2} & \Xi_{2a2a} & \Xi_{2a2b} \\ \Xi_{2b1} & \Xi_{2b1a} & \Xi_{2b2} & \Xi_{2b2a} & \Xi_{2b2b} \end{bmatrix}. \tag{S3}$$

Only the matrix elements in the coarse grid are explicitly calculated (those highlighted in bold), e.g., $\mathbf{\Xi_{11}}$. The matrix elements involving at least one transition in the fine grid, will be approximated to either zero or the corresponding matrix element in the

coarse grid. This will depend on the element in question and the approach taken (DKE or FKE).

Bear in mind that these “matrix elements” are 1-by-1 matrices, which stems from the fact that we initially considered only one coarse-grid transition per group. Otherwise, these ”matrix elements” would be n-by-m matrices, where n and m are the sizes of the two *groups* forming that *block*, i.e., the number of transitions on each of those *groups*.

C. Diagonal Kernel Extension (DKE)

In this approximation, we set to zero some of the matrix elements according to Eq. 12 of the manuscript. This implies, for instance, $\Xi_{11a} = \Xi_{1a1} = 0$ while $\Xi_{1a1a} = \mathbf{\Xi_{11}}$. This approximation leads to a matrix of the form

$$\begin{bmatrix} \mathbf{\Xi_{11}} & \Xi_{11a} & \mathbf{\Xi_{12}} & \Xi_{12a} & \Xi_{12b} \\ \Xi_{1a1} & \Xi_{1a1a} & \Xi_{1a2} & \Xi_{1a2a} & \Xi_{1a2b} \\ \mathbf{\Xi_{21}} & \Xi_{21a} & \mathbf{\Xi_{22}} & \Xi_{22a} & \Xi_{22b} \\ \Xi_{2a1} & \Xi_{2a1a} & \Xi_{2a2} & \Xi_{2a2a} & \Xi_{2a2b} \\ \Xi_{2b1} & \Xi_{2b1a} & \Xi_{2b2} & \Xi_{2b2a} & \Xi_{2b2b} \end{bmatrix} \rightarrow \begin{bmatrix} \mathbf{\Xi_{11}} & 0 & \mathbf{\Xi_{12}} & 0 & 0 \\ 0 & \Xi_{1a1a} & 0 & \Xi_{1a2a} & 0 \\ \mathbf{\Xi_{21}} & 0 & \mathbf{\Xi_{22}} & 0 & 0 \\ 0 & \Xi_{2a1a} & 0 & \Xi_{2a2a} & 0 \\ 0 & 0 & 0 & 0 & \Xi_{2b2b} \end{bmatrix}. \tag{S4}$$

The matrix-vector multiplication then leads to

$$\begin{bmatrix} \mathbf{\Xi_{11}} & 0 & \mathbf{\Xi_{12}} & 0 & 0 \\ 0 & \mathbf{\Xi_{11}} & 0 & \mathbf{\Xi_{12}} & 0 \\ \mathbf{\Xi_{21}} & 0 & \mathbf{\Xi_{22}} & 0 & 0 \\ 0 & \mathbf{\Xi_{21}} & 0 & \mathbf{\Xi_{22}} & 0 \\ 0 & 0 & 0 & 0 & \mathbf{\Xi_{22}} \end{bmatrix} \cdot \begin{bmatrix} \mathbf{Vi_1} \\ Vi_{1a} \\ \mathbf{Vi_2} \\ Vi_{2a} \\ Vi_{2b} \end{bmatrix} \approx \begin{bmatrix} \mathbf{Vo_1} \\ Vo_{1a} \\ \mathbf{Vo_2} \\ Vo_{2a} \\ Vo_{2b} \end{bmatrix}. \tag{S5}$$

The output vector would then be composed as

$$\begin{bmatrix} \mathbf{Vo_1} \\ Vo_{1a} \\ \mathbf{Vo_2} \\ Vo_{2a} \\ Vo_{2b} \end{bmatrix} \approx \begin{bmatrix} \mathbf{\Xi_{11}} \cdot \mathbf{Vi_1} + \mathbf{\Xi_{12}} \cdot \mathbf{Vi_2} \\ \mathbf{\Xi_{11}} \cdot Vi_{1a} + \mathbf{\Xi_{12}} \cdot Vi_{2a} \\ \mathbf{\Xi_{21}} \cdot \mathbf{Vi_1} + \mathbf{\Xi_{22}} \cdot \mathbf{Vi_2} \\ \mathbf{\Xi_{21}} \cdot Vi_{1a} + \mathbf{\Xi_{22}} \cdot Vi_{2a} \\ \mathbf{\Xi_{22}} \cdot Vi_{2b} \end{bmatrix} . \tag{S6}$$

Note that, in general (i.e., except in the very first Haydock iteration), $\mathbf{Vi_1} \neq Vi_{1a}$ due to the energy shifts coming from the diagonal part of the excitonic Hamiltonian.

This approximation then results in a very simple implementation, as it can be reformulated as

$$\begin{bmatrix} \mathbf{\Xi_{11}} & \mathbf{\Xi_{12}} \\ \mathbf{\Xi_{21}} & \mathbf{\Xi_{22}} \end{bmatrix} \cdot \begin{bmatrix} \mathbf{Vi_1} & Vi_{1a} & 0 \\ \mathbf{Vi_2} & Vi_{2a} & Vi_{2b} \end{bmatrix} = \begin{bmatrix} \mathbf{Vo_1} & Vo_{1a} & {\color{red}0} \\ \mathbf{Vo_2} & Vo_{2a} & Vo_{2b} \end{bmatrix} . \tag{S7}$$

This allows us to always work with the same BSE kernel matrix as in the coarse grid. Moreover, the structure of the Haydock subroutine can be somewhat maintained, as these double-grid extended Haydock vectors can be folded into “Haydock matrices”. Then, what is a matrix-vector multiplication in single-grid Haydock, becomes a matrix-matrix multiplication in double-grid Haydock. The number 0 marked in red in Eq. S7 has to be imposed in the code, i.e. preventing that matrix element from being calculated as the result would not be zero.

A noteworthy aspect of the DKE approximation is that index a (or $i = 2$ in the nomenclature of Eq. 12 of the manuscript) will not necessarily mean the same for $\mathbf{I} = 1$ and $\mathbf{I}' = 2$. In other words, the fine-grid point κ_{1_a} will not necessarily be at the same relative position from $\mathbf{k_1}$ as κ_{2_a} is from $\mathbf{k_2}$ (although the opposite will be true if $\mathbf{I} = \mathbf{I}'$ because they are essentially the same $\mathbf{k}$-points). Hence, the decision to couple κ_{1_a} with κ_{2_a} (instead of κ_{2_b}) is ultimately an arbitrary choice, but one maintains the approximated BSE kernel also sharply peaked at $\mathbf{q} = \mathbf{0}$. Let us analyse the case $\mathbf{I} = \mathbf{I}'$, in which the corresponding matrix elements belong to the diagonal (e.g., $\mathbf{\Xi_{22}}$). If we were to couple a with b, we would have a $\mathbf{q} \neq \mathbf{0}$, off-diagonal matrix element ($\Xi_{2_a 2_b}$)

with the same value as the corresponding diagonal $\mathbf{q} = \mathbf{0}$ matrix element ($\mathbf{\Xi_{22}}$), which would introduce artefacts in the optical response. This issue is more subtle when it comes to the $\mathbf{I} \neq \mathbf{I}'$ matrix elements, which are off-diagonal (e.g., $\mathbf{\Xi_{12}}$). In this case, there would be no obvious choice as to whether a should be coupled with a or b. Any attempt to decide this based on the relative positions of those $\mathbf{k}$-points would make the method heavily dependent on the shape of the $\mathbf{k}$-grids used as input, and probably less robust. Ultimately, our double-grid method relies on these off diagonal elements being small compared to diagonal ones, as we are only interested in the latter. This is another way to see the limitations this method faces when the BSE kernel is not sharply peaked at $\mathbf{q} = \mathbf{0}$. Moreover, this is in line with the fact that this method cannot work for systems with strongly bound excitons, as these usually imply important contributions from $\mathbf{q} \neq \mathbf{0}$ matrix elements.

D. Full Kernel Extension (FKE)

In this approximation, we consider that all matrix elements involving transitions in the fine grid will be equal to the corresponding ones in the coarse grid, according to Eq. 16 of the manuscript. This approximation leads to a matrix of the form

$$\begin{bmatrix} \mathbf{\Xi_{11}} & \Xi_{11a} & \mathbf{\Xi_{12}} & \Xi_{12a} & \Xi_{12b} \\ \Xi_{1a1} & \Xi_{1a1a} & \Xi_{1a2} & \Xi_{1a2a} & \Xi_{1a2b} \\ \mathbf{\Xi_{21}} & \Xi_{21a} & \mathbf{\Xi_{22}} & \Xi_{22a} & \Xi_{22b} \\ \Xi_{2a1} & \Xi_{2a1a} & \Xi_{2a2} & \Xi_{2a2a} & \Xi_{2a2b} \\ \Xi_{2b1} & \Xi_{2b1a} & \Xi_{2b2} & \Xi_{2b2a} & \Xi_{2b2b} \end{bmatrix} \rightarrow \begin{bmatrix} \mathbf{\Xi_{11}} & \mathbf{\Xi_{11}} & \mathbf{\Xi_{12}} & \mathbf{\Xi_{12}} & \mathbf{\Xi_{12}} \\ \mathbf{\Xi_{11}} & \mathbf{\Xi_{11}} & \mathbf{\Xi_{12}} & \mathbf{\Xi_{12}} & \mathbf{\Xi_{12}} \\ \mathbf{\Xi_{21}} & \mathbf{\Xi_{21}} & \mathbf{\Xi_{22}} & \mathbf{\Xi_{22}} & \mathbf{\Xi_{22}} \\ \mathbf{\Xi_{21}} & \mathbf{\Xi_{21}} & \mathbf{\Xi_{22}} & \mathbf{\Xi_{22}} & \mathbf{\Xi_{22}} \\ \mathbf{\Xi_{21}} & \mathbf{\Xi_{21}} & \mathbf{\Xi_{22}} & \mathbf{\Xi_{22}} & \mathbf{\Xi_{22}} \end{bmatrix}. \tag{S8}$$

Consequently, the matrix vector multiplication would be

$$\begin{bmatrix} \mathbf{\Xi_{11}} & \mathbf{\Xi_{11}} & \mathbf{\Xi_{12}} & \mathbf{\Xi_{12}} & \mathbf{\Xi_{12}} \\ \mathbf{\Xi_{11}} & \mathbf{\Xi_{11}} & \mathbf{\Xi_{12}} & \mathbf{\Xi_{12}} & \mathbf{\Xi_{12}} \\ \mathbf{\Xi_{21}} & \mathbf{\Xi_{21}} & \mathbf{\Xi_{22}} & \mathbf{\Xi_{22}} & \mathbf{\Xi_{22}} \\ \mathbf{\Xi_{21}} & \mathbf{\Xi_{21}} & \mathbf{\Xi_{22}} & \mathbf{\Xi_{22}} & \mathbf{\Xi_{22}} \\ \mathbf{\Xi_{21}} & \mathbf{\Xi_{21}} & \mathbf{\Xi_{22}} & \mathbf{\Xi_{22}} & \mathbf{\Xi_{22}} \end{bmatrix} \cdot \begin{bmatrix} \mathbf{Vi_1} \\ Vi_{1a} \\ \mathbf{Vi_2} \\ Vi_{2a} \\ Vi_{2b} \end{bmatrix} \approx \begin{bmatrix} \mathbf{Vo_1} \\ Vo_{1a} \\ \mathbf{Vo_2} \\ Vo_{2a} \\ Vo_{2b} \end{bmatrix}. \tag{S9}$$

The output vector would then be composed as

$$\begin{bmatrix} \mathbf{Vo_1} \\ Vo_{1a} \\ \mathbf{Vo_2} \\ Vo_{2a} \\ Vo_{2b} \end{bmatrix} \approx \begin{bmatrix} \mathbf{\Xi_{11}} \cdot (\mathbf{Vi_1} + Vi_{1a}) + \mathbf{\Xi_{12}} \cdot (\mathbf{Vi_2} + Vi_{2a} + Vi_{2b}) \\ \mathbf{\Xi_{11}} \cdot (\mathbf{Vi_1} + Vi_{1a}) + \mathbf{\Xi_{12}} \cdot (\mathbf{Vi_2} + Vi_{2a} + Vi_{2b}) \\ \mathbf{\Xi_{21}} \cdot (\mathbf{Vi_1} + Vi_{1a}) + \mathbf{\Xi_{22}} \cdot (\mathbf{Vi_2} + Vi_{2a} + Vi_{2b}) \\ \mathbf{\Xi_{21}} \cdot (\mathbf{Vi_1} + Vi_{1a}) + \mathbf{\Xi_{22}} \cdot (\mathbf{Vi_2} + Vi_{2a} + Vi_{2b}) \\ \mathbf{\Xi_{21}} \cdot (\mathbf{Vi_1} + Vi_{1a}) + \mathbf{\Xi_{22}} \cdot (\mathbf{Vi_2} + Vi_{2a} + Vi_{2b}) \end{bmatrix} . \tag{S10}$$

It is important to note that $\mathbf{Vo_1} = Vo_{1a}$ and $\mathbf{Vo_2} = Vo_{2a} = Vo_{2b}$. Despite the computational advantage of not having to calculate all output vectors, this is essentially a drawback of the FKE approach (see below). Also, note that, unlike the DKE approach, FKE requires the kernel to be scaled down by a factor relative to the number of coarse-grid and fine-grid **k**-points.

E. Further analysis of kernel extension to the fine grid

In order to further explain the better performance of DKE over FKE, let us now consider a system with several **k**-points and only one pair of bands. In this case, the e-h space is composed directly by one vertical transition at each **K**- or κ-point, and the sharply peaked matrix elements correspond to those in the diagonal ($\mathbf{q} = \mathbf{0}$), while the rest of the matrix is considerably sparse. Nonetheless, this does not necessarily mean that the BSE kernel matrix meets the criteria for being strictly diagonally dominant (i.e., a matrix where all elements $A_{i,j}$ satisfy $|A_{i,i}| > \sum_{j \neq i} |A_{i,j}|$ for all i). In fact, this is not the case. Even in the simple example of monolayer MoS_2 with only one pair of bands, the off-diagonal elements of the BSE kernel, albeit very small, add up to a value that is higher than the diagonal element in many rows of the matrix. Admittedly, the situation changes if one considers the two-particle Hamiltonian, where the energies are added to the diagonal. Indeed, the two-particle Hamiltonian is strictly diagonal dominant in the case of this two-band monolayer MoS_2 example. Alternatively, we can consider only a given *block* of the BSE kernel, determined by two *fragments* of the e-h basis set. We will consider the blocks in the

diagonal, which are square by construction and contain the $\mathbf{q} = 0$ matrix elements we are discussing. Each of this blocks also satisfies the condition for being strictly diagonal dominant. The determination of the *fragments* is somewhat arbitrary and only relevant to Yambo, but it nonetheless helps us visualise the problem at hand. For instance, Fig. S8 displays the real part of one block of the BSE kernel (Eq. 3 of the manuscript) for two-band monolayer MoS_2. Fig. S8A shows the coarse grid BSE kernel block (i.e., our starting point in the double grid approach) while Fig. S8B and C display the fine grid BSE kernels approximated by DKE and FKE, respectively. Finally, Fig. S8D illustrates the *full* BSE kernel as calculated with a $12 \times 12 \times 1$ $\mathbf{k}$-grid. The term *full* here refers to the BSE kernel that is obtained if the $\mathbf{k}$-grid that normally acts as a fine (double) grid, is actually used as the single $\mathbf{k}$-grid in a BSE calculation with no double grid approach. In other words, Fig. S8D is the matrix we are trying to approximate (which *can* be calculated in full in this case as only a $12 \times 12 \times 1$ $\mathbf{k}$-grid is being used). Importantly, we see that both blocks in panels A and D are nearly diagonal. Now, it becomes apparent from panel B that the DKE approach respects the diagonally-dominant nature of the block in panel A, and thus represents a better approximation to the *full* block in panel D. At variance, the FKE approach prevents, by construction, any matrix from being diagonally dominant. Indeed, the FKE kernel approaches a block-diagonal matrix, which represents a greater deviation from the *full* block in panel D, and hence, a worse approximation to it.

We now consider a more realistic scenario where we include various conduction and valence bands in the BSE calculation in order to achieve convergence. In this case, the two-particle Hamiltonian is not diagonally dominant any more. This is because the e-h space grows considerably and so does the sum of non-diagonal elements in a given row, which ends up overtaking the value of the diagonal element. In other words, adding the transition energies to the diagonal of the BSE kernel (see Eq. 6 of the manuscript) is no longer enough to ensure the diagonally dominant character of the two-particle Hamiltonian when many bands are considered, unlike the two-band system described above. Nonetheless, we can still use arguments of this nature to

address the comparison between DKE and FKE. Let us define the diagonal dominance per row i as $dd_i = \frac{\sum_{j\neq i}|H^{2p}_{i,j}|}{|H^{2p}_{i,i}|}$. Fig. S9 shows dd_i of all rows for the case of MoS_2 with all the bands required for convergence. As in Fig. S8, DKE and FKE represent the matrices calculated by extending a $6 \times 6 \times 1$ coarse $\mathbf{K}$-grid Hamiltonian into a $12\times12\times1$ double κ-grid one, while the fine grid data points correspond to the matrix we are trying to approximate, i.e, the Hamiltonian obtained with one single grid of $12 \times 12 \times 1$ $\mathbf{k}$-points. We can immediately confirm that none of these two-particle Hamiltonians are diagonally dominant any more, as they have rows with $dd_i > 1$. In addition, an average dd_i over all rows for each case is shown with flat lines in Fig. S9. It becomes apparent that the DKE and fine grid matrices have very similar average dd_i, while that of the FKE matrix is an order of magnitude higher. In other words, the DKE method is better than FKE at extending the coarse grid two-particle Hamiltonian into a matrix that closely resembles that of the fine grid Hamiltonian from the viewpoint of diagonal dominance.

Finally, there is an algebraic argument against the FKE approach that can be derived from Eqs. S9-S10 (or Eq. 17 in the manuscript), in comparison with their DKE analogues, Eqs. S5-S6 (or Eq. 15 in the manuscript). First, it should be considered that these equations are embedded in a loop of Haydock iterations. As part of this loop, the Haydock vectors are multiplied by the two-particle Hamiltonian H^{2p}, which entails the multiplications in Eqs. S6 or S10 plus a shift due to the diagonal matrix of transition energies (see Eq. 6 in the manuscript). Associated to this multiplication, there is a subtle point about the way in which the DKE or FKE approaches handle the benefits that the double grid brings. The impact of the double grid in the final spectrum depends crucially on the diagonal matrix of transition energies $E_{nm\mathbf{k}}\ \delta_{nn'}\delta_{mm'}\delta_{\mathbf{k}\mathbf{k}'}$. In other words, given that the kernel in the fine grid is approximated as equal to that of the coarse grid, the shift $E_{nm\mathbf{k}}\ \delta_{nn'}\delta_{mm'}\delta_{\mathbf{k}\mathbf{k}'}$ is what makes transitions inside a given domain $\mathbf{Dom}(\mathbf{K_I})$ different among themselves. Hence, this *differential shift* is why the double grid method gives a different spectrum than the coarse grid alone. As mentioned before, in the very first Haydock iteration,

the components of the Haydock vector in the coarse and fine grid are the same, as they are equally initialised (see Eq. 13 of the manuscript). However, in the second iteration and beyond, this vector will have picked up a shift coming from the diagonal matrix of transition energies, which will differ among components in the coarse and fine grid. **This** is the impact of the double grid in the Haydock method (since the fine grid kernel is approximated by the coarse grid). However, this impact is somewhat *lost* or *averaged out* in the FKE vector-matrix multiplication (Eq. 17 or S9-S10). This effect is exemplified in Eq. S10, where $\mathbf{Vo_1} = Vo_{1a}$ and $\mathbf{Vo_2} = Vo_{2a} = Vo_{2b}$. In essence, Eq. 17 (or S10) tells us that $r_{nm\kappa_{\mathbf{I}_i}}$ (or Vo_{1i}) will be equal $\forall i \in \mathbf{Dom}(\mathbf{K_I})$, no matter how different $c_{n'm'\kappa_{\mathbf{I'}_{i'}}}$ (or $Vi_{I'i'}$) may be across different values of I' and i'. Importantly, this difference among values of $c_{n'm'\kappa_{\mathbf{I'}_{i'}}}$ (or $Vi_{I'i'}$) is a result of the differential shift *gained* in the previous Haydock iteration, so Eq. 17 (or S10) effectively causes a *reset* of the Haydock vector that enters the loop at each iteration. Of course, these output components $r_{nm\kappa_{\mathbf{I}_i}}$ (equal among themselves) are different from ('better' than) the ones that would have been obtained if the input components $c_{n'm'\kappa_{\mathbf{I'}_{i'}}}$ had not been different at all. But overall, the FKE hinders the ability of the double grid to have an impact in the Haydock output vectors via the diagonal matrix of transition energies in the excitonic Hamiltonian. This detrimental *'homogenisation'* of the output vectors is not present in the DKE approach, which lets the benefits of the double grid (the differential shifts) accumulate over successive iterations, leading to a greater impact of the fine grid overall, and a better final spectrum.

---

[1] Albrecht, S., Reining, L., Del Sole, R. & Onida, G. Ab initio calculation of excitonic effects in the optical spectra of semiconductors. *Physical Review Letters* **80**, 4510 (1998).

[2] Albrecht, S., Reining, L., Del Sole, R. & Onida, G. Response to comment. *Physical Review Letters* **83**, 3971 (1999).

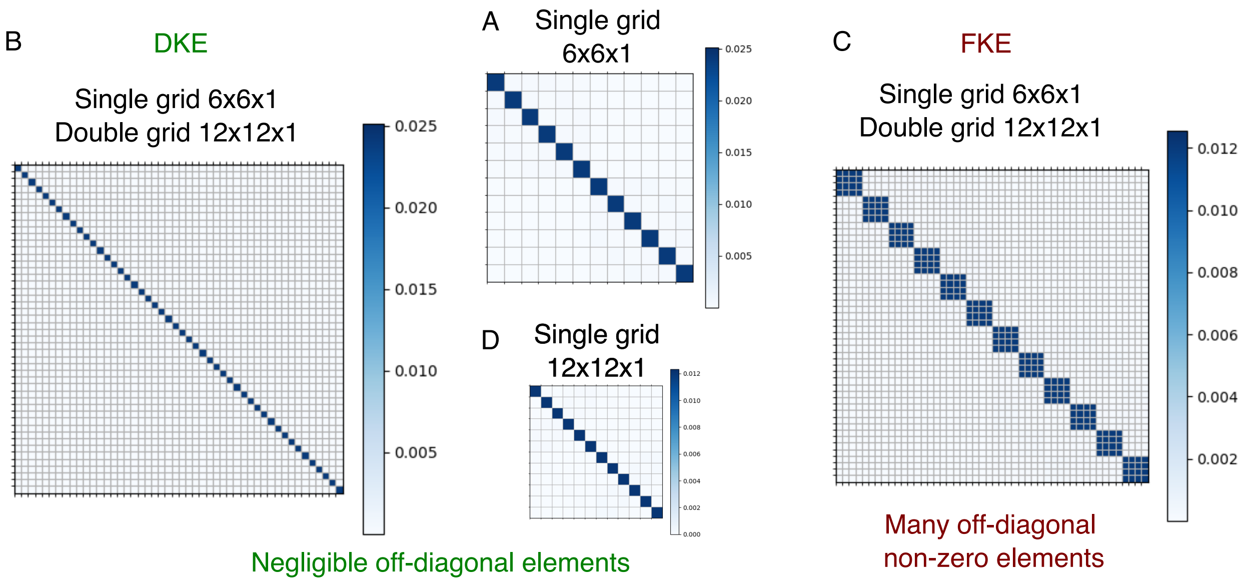

FIG. S8: Comparison of the BSE kernel matrices obtained via diagonal kernel extension (DKE) and full kernel extension (FKE). The data plotted here is the real part of one *block* of the BSE kernel in MoS_2 with only one pair of bands.

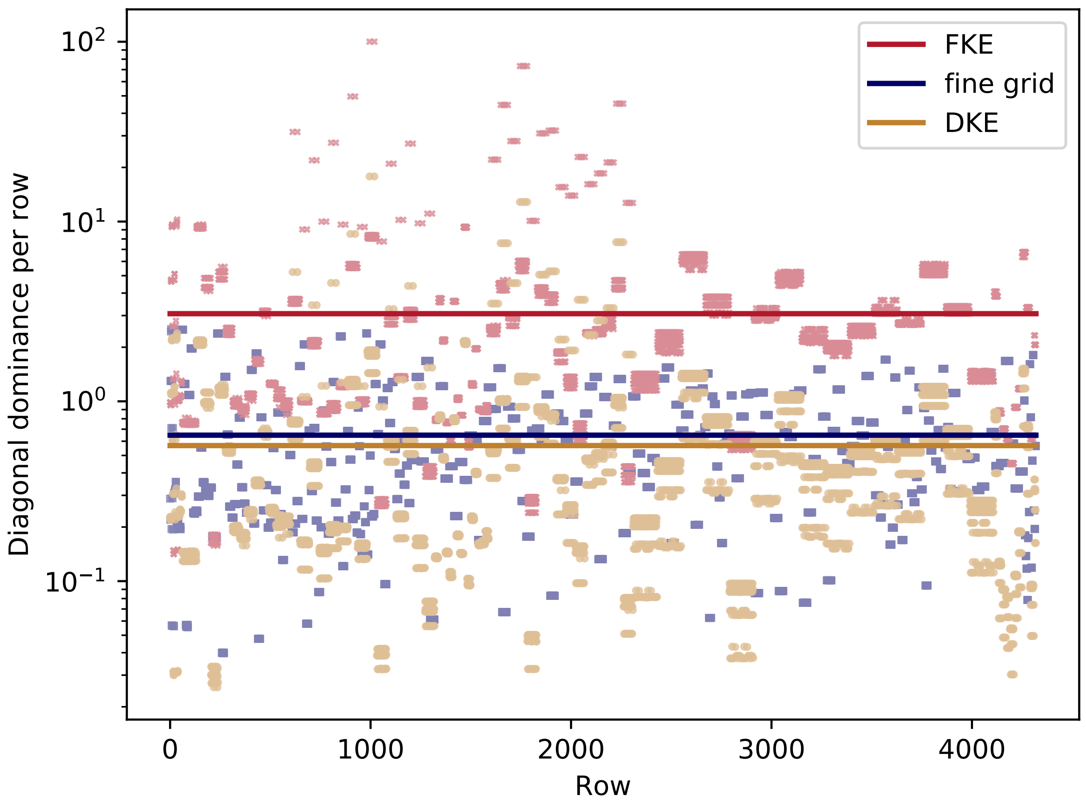

FIG. S9: Diagonal dominance of the two-particle Hamiltonian matrices obtained via diagonal kernel extension (DKE) and full kernel extension (FKE), compared with the case of . The data plotted here corresponds to MoS_2 with all the bands required for convergence. The DKE and FKE matrices are obtained from a $6 \times 6 \times 1$ coarse $\mathbf{K}$-grid and a $12 \times 12 \times 1$ double κ-grid. The fine grid data is simply the matrix that DKE and FKE try to approximate, i.e., the two-particle Hamiltonian obtained with one single grid of $12 \times 12 \times 1$ $\mathbf{k}$-points.
